# Dietary supplementation of blend of organic acids and monoglycerides alleviated diarrhea and systemic inflammation of weaned pigs experimentally infected with enterotoxigenic *Escherichia coli* F18

**DOI:** 10.1186/s40104-024-01148-8

**Published:** 2025-01-22

**Authors:** Sangwoo Park, Shuhan Sun, Supatirada Wongchanla, Ying Chen, Xunde Li, Yanhong Liu

**Affiliations:** 1 Department of Animal Science, University of California, Davis, CA 95616 USA; 2 Animal Nutrition, Eastman Chemical Company, Kingsport, TN USA; 3 School of Veterinary Medicine, University of California, Davis, CA 95616 USA

**Keywords:** Acidifiers, Antimicrobial agents, Diarrhea, Enterotoxigenic *Escherichia coli*, Monoglycerides, Systemic immunity, Weaned pigs

## Abstract

**Background:**

The emergence of antibiotic resistant microorganisms associated with conventional swine production practices has increased interest in acid-based compounds having antimicrobial properties and other biological functions as nutritional interventions. Despite the interest in organic acids and monoglycerides, few studies have examined the effects of the combination of these acid-based additives in weaned pigs under disease challenge conditions. Therefore, this study aimed to investigate the effects of dietary supplementation with blend of organic acids and/or medium-chain fatty acid monoglycerides on intestinal health and systemic immunity of weaned pigs experimentally infected with an enterotoxigenic *Escherichia coli* (ETEC) F18 at 4-week of age.

**Results:**

Dietary supplementation of organic acids, monoglycerides, or both organic acids and monoglycerides (combination) reduced (*P* < 0.05) the diarrhea frequency of ETEC F18-infected pigs throughout the experimental period (d −7 to 21 post-inoculation). This is consistent with the reduced (*P* < 0.05) proportion of β-hemolytic coliforms in feces observed for the organic acid and combination treatments on d 10 post-inoculation. Supplementation of organic acids, monoglycerides, or combination also reduced (*P* < 0.05) bacterial translocation in mesenteric lymph nodes on d 21 post-inoculation. Pigs fed with monoglycerides or combination had lower (*P* < 0.05) white blood cells on d 5 post-inoculation, and pigs fed the combination also had lower (*P* < 0.05) lymphocytes than pigs in control group. Monoglyceride supplementation increased (*P* < 0.05) white blood cells and neutrophils compared with control group on d 14 post-inoculation. However, supplementation with organic acid blend, monoglyceride blend, or combination did not affect growth performance in this experiment.

**Conclusions:**

Supplementation with monoglycerides or organic acids alone or in combination improves the detrimental effects of ETEC F18 infection in weaned pigs, as indicated by reduced diarrhea, fecal shedding of β-hemolytic coliforms, and bacterial translocation, and thus enhancing disease resistance. Monoglycerides reduced the inflammatory response during peak infection, but their immunomodulatory and possible synergistic effects with organic acids need to be further investigated.

## Background

Weaning, a critical and inevitable stage in pig production, presents a variety of challenges, including rapid dietary transitions, immature physiological and immune systems, and environmental changes [1, 2]. This multifactorial stressor decreases appetite, induces intestinal dysfunction, and increases susceptibility to pathogens in weaned pigs, resulting not only in post-weaning diarrhea but also in an enhanced inflammatory response [3]. Colonization of enterotoxigenic *Escherichia coli* (ETEC) in the small intestine, especially strains expressing the F18 fimbriae, is the most common cause of post-weaning diarrhea in pigs [4]. The changes in the host resulting from interaction with ETEC F18 are not fully understood, but disturbances in fluid secretion and electrolyte imbalances caused by virulence factors have been reported [5, 6]. The ETEC F18 infection in weaned pigs leads to diarrhea, dehydration, stunted growth, and even significant mortality [7, 8]. Moreover, stress in the early life stage can exacerbate the severity and duration of this enteric disease and vice versa [9].

Nutritional strategies to manage the adverse effects of weaning stress and post-weaning diarrhea have become even more important, particularly as usage of antibiotic growth promoters has been restricted and pharmaceutical levels of ZnO have been banned in Europe due to increasing concerns over public health risks from antimicrobial resistance and environmental footprint [10–12]. Organic acids, organic compounds containing a carboxyl group, have been proposed as a viable alternative to antibiotic growth promoters due to their benefits on animal health and growth [13]. During the weaning period, pigs have immature digestive systems and produce insufficient hydrochloric acid in the stomach, making supplementation with organic acids particularly significant [14]. For example, organic acid supplementation can lower the pH of the gastric content, thereby increasing pepsin activity and reducing pathogen load by creating a hostile environment for pathogens [15]. The benefits of organic acid supplementation extend beyond these effects, encompassing a variety of bioactivities throughout the gastrointestinal tract: 1) reducing digesta passage rate and improving nutrient digestibility; 2) enhancing mucosal integrity and function by providing energy and nutrients; and 3) inhibiting pathogenic bacterial growth and mitigating post-weaning diarrhea, ultimately improving nutrient utilization and growth performance in pigs [14, 16, 17]. Although organic acids have been used in swine feed for a while, inconsistent responses in animals have highlighted the need for further research to explore complementary strategies, such as organic acid blends or organic acid derivatives, to enhance the efficacy [12, 13, 18].

Monoglycerides, glycerol monoesters of organic fatty acids, have attracted interest as an alternative to corresponding fatty acids. Esterification of organic fatty acids with glycerol increases stability, reduces unpleasant odors, and allows for a gradual release of the active substance throughout the intestine, facilitating their use as additives [19]. Monoglycerides of medium-chain fatty acids (MCFA; C6–12) have demonstrated antibacterial activities against a broad spectrum of pathogens, including *Escherichia coli* [18, 20, 21], and are classified as GRAS (Generally Recognized As Safe) in the United States and widely utilized in the food industry [22]. Monoglycerides of MCFA have been reported to play various biological roles, such as immunomodulation [23, 24] and improvement of intestinal health [25–27], based on in vitro and in vivo experiments. In addition, synergistic antibacterial effects have been observed when MCFA or their esters were combined with organic acids [28, 29]. Despite the interest in organic acids and monoglycerides and their potential synergy, few studies have explored their use as blends to enhance disease resistance and resilience in pigs. Therefore, the objectives of the current study were 1) to evaluate the influence of dietary supplementation of organic acids or monoglycerides on the growth performance, intestinal health, and immune responses of weaned pigs experimentally infected with ETEC F18; and 2) to investigate whether combining these acid-based additives has synergistic effects on animal health in this in vivo challenge model.

## Materials and methods

### Animals, housing, experimental design, and diet

The protocol for this study was reviewed and approved by the Institutional Animal Care and Use Committee at the University of California, Davis (IACUC #21875). A total of 40 piglets (initial body weight [BW] = 7.81 ± 0.84 kg) weaned around 21 days of age were obtained from the Swine Teaching and Research Center at the University of California, Davis. The 10 sows and their piglets used in this experiment did not receive *Escherichia coli* vaccines, antibiotic injections, or antibiotics in creep feed. Before weaning, fecal samples were collected from sows and all their piglets destined for this study, to verify the absence of β-hemolytic *Escherichia coli*. The *Escherichia coli* F18 receptor status was also tested based on the methods of Kreuzer et al. [30], and piglets susceptible to *Escherichia coli* F18 were selected for this study. After weaning, all pigs were randomly assigned to one of the four dietary treatments (10 replicates/treatment) in a randomized complete block design with weaning BW as block and pig as the experimental unit. Pigs were individually housed in pens (0.61 m × 1.22 m) for 28 d after weaning, including 7 d before and 21 d after the *Escherichia coli* challenge. All piglets had free access to feed and water. The light was on at 07:30 h and off at 19:30 h daily in the environmental control unit.

The four dietary treatments included: 1) a corn-soybean meal-based nursery basal diet (control); 2) the basal diet with 0.3% organic acid blend (Acitra G20C, Eastman, blend of organic acids including formic acid); 3) the basal diet with 0.3% monoglyceride blend (Entero-Nova 410C, Eastman, blend of short- and medium-chain fatty acids); and 4) the basal diet with Acitra G20C at 0.2% and Entero-Nova 410C at 0.2% (combination). A 2-phase feeding program was used, with the first 2 weeks as phase 1 and the last 2 weeks as phase 2 (Table 1). The basal diet did not include spray-dried plasma, high levels of zinc oxide (exceeding recommendation and normal practice), and antibiotics. All diets were formulated to meet pig nutritional requirements [31] and provided as mash form throughout the experiment.

**Table 1:** Ingredient compositions of experimental diets^a^

Ingredient, %	Control (phase 1)	Control (phase 2)
Corn	44.70	54.29
Dried whey	15.00	10.00
Soybean meal	21.50	30.50
Fish meal	3.00	-
Lactose	6.00	-
Soy protein concentrate	5.00	-
Soybean oil	2.00	2.00
Limestone	0.98	1.00
Dicalcium phosphate	0.55	0.90
L-Lysine·HCl	0.34	0.39
DL-Methionine	0.14	0.12
L-Threonine	0.09	0.10
Salt	0.40	0.40
Vit-mineral^b^	0.30	0.30
Total	100.00	100.00
Calculated energy and nutrient		
Metabolizable energy, kcal/kg	3,418	3,375
Net energy, kcal/kg	2,564	2,508
Crude protein, %	20.84	20.19
Arg,^c^ %	1.20	1.19
His,^c^ %	0.48	0.48
Ile,^c^ %	0.80	0.77
Leu,^c^ %	1.57	1.53
Lys,^c^ %	1.35	1.29
Met,^c^ %	0.44	0.40
Thr,^c^ %	0.79	0.76
Trp,^c^ %	0.23	0.23
Val,^c^ %	0.86	0.82
Met + Cys,^c^ %	0.74	0.71
Phe + Tyr,^c^ %	1.44	1.42
Ca, %	0.80	0.75
Total P, %	0.62	0.61
Digestible P, %	0.40	0.37
Analyzed nutrients, %		
Dry matter	92.00	90.40
Crude protein	23.00	22.70
Acid detergent fiber	2.80	3.60
Neutral detergent fiber	6.40	8.40
Total Ca	1.39	1.48
Total P	0.64	0.65

^a^In each phase, three additional diets were formulated by adding 0.3% organic acids, 0.3% monoglycerides, or blend of 0.2% organic acids and 0.2% monoglycerides to the control diet, respectively

^b^Provided by the United Animal Health (Sheridan, IN, USA). Provided the following quantities of vitamins and micro minerals per kilogram of complete diet: vitamin A as retinyl acetate, 11,136 IU; vitamin D_3_ as cholecalciferol, 2,208 IU; vitamin E as DL-alpha-tocopheryl acetate, 66 IU; vitamin K as menadione dimethylpyrimidinol bisulfite, 1.42 mg; thiamin as thiamine mononitrate, 0.24 mg; riboflavin, 6.59 mg; pyridoxine as pyridoxine hydrochloride, 0.24 mg; vitamin B_12_, 0.03 mg; D-pantothenic acid as D-calcium pantothenate, 23.5 mg; niacin, 44.1 mg; folic acid, 1.59 mg; biotin, 0.44 mg; Cu, 20 mg as copper sulfate and copper chloride; Fe, 126 mg as ferrous sulfate; I, 1.26 mg as ethylenediamine dihydriodide; Mn, 60.2 mg as manganese sulfate; Se, 0.3 mg as sodium selenite and selenium yeast; and Zn, 125.1 mg as zinc sulfate

^c^Amino acids were indicated as standardized ileal digestible amino acids

After 7-day adaptation period, all pigs were orally inoculated with 3 mL of ETEC F18 for three consecutive days from d 0 post-inoculation (PI). The ETEC F18 was originally isolated from a field disease outbreak by the University of Montreal (isolate number: ECL22131). The ETEC F18 expresses heat-labile and heat-stable toxins "a" and "b". The inoculums were prepared at 10^10^ colony-forming units (CFU) per 3 mL dose in phosphate-buffered saline. This dose caused mild diarrhea in the current study, consistent with our previously published research [32–34].

### Clinical observations and sample collections

The procedures of this experiment were adapted from previous research [32, 35–37]. Clinical observations (fecal score and alertness score) were recorded twice daily throughout the study. The fecal score of each pig was assessed each day visually by two independent evaluators, with the score ranging from 1 to 5 (1 = normal feces, 2 = moist feces, 3 = mild diarrhea, 4 = severe diarrhea, and 5 = watery diarrhea). The frequency of diarrhea was calculated as the percentage of the pig days with fecal score of 3 or greater. Alertness was scored from 1 to 3 (1 = normal, 2 = slightly depressed or listless, and 3 = severely depressed or recumbent). Scores for alertness did not exceed two throughout the experiment (data not shown).

Pigs were weighed and feed intake was measured on weaning day (d −7; initial BW), d 0 (before first inoculation), 7, 14, and 21 PI. Average daily gain (ADG), average daily feed intake (ADFI), and feed efficiency (gain:feed ratio) were calculated for each period. Fecal samples were collected from the rectum of all pigs on d −7, 0, 2, 5, 7, 10, 14, and 21 PI using a cotton swab to test β-hemolytic coliforms and the percentage of β-hemolytic coliforms to total coliforms (see details below) [32, 35–37]. Blood samples were collected from the jugular vein of all pigs with or without ethylenediaminetetraacetic acid to yield whole blood and serum, respectively, before ETEC challenge (d 0), and on d 2, 5, or 14 PI. Serum samples were collected and immediately stored at −80 °C before further analysis.

All pigs were euthanized at the end of the experiment. Before euthanization, pigs were anesthetized with 1 mL mixture of 100 mg telazol, 50 mg ketamine, and 50 mg xylazine (2:1:1) by intramuscular injection. After anesthesia, intracardiac injection with 78 mg Fatal-Plus solution (sodium pentobarbital, MWI Animal Health, Visalia, CA, USA) per 1 kg of BW was used to euthanize each pig. Mesenteric lymph nodes were aseptically collected and then pooled within the pig, ground, diluted, and plated on brain heart infusion agar for measurement of total bacteria, and the results were expressed as CFU/g of mesenteric lymph nodes [38, 39]. Spleen samples were analyzed in the same manner as mesenteric lymph nodes for bacterial translocation.

### Detection of β-hemolytic coliforms

Briefly, fecal samples were plated on Columbia blood agar with 5% sheep blood to identify hemolytic coliforms, which can lyse red blood cells (RBC) surrounding the colony. Fecal samples were also plated on MacConkey agar to enumerate total coliforms. Hemolytic colonies from the blood agar were sub-cultured on MacConkey agar to confirm that they were lactose-fermenting bacteria and flat pink colonies. All plates were incubated at 37 °C for 24 h in an air incubator. Populations of both total coliforms and β-hemolytic coliforms on blood agar were visually scored from 0 to 8 (0 = no bacterial growth, 8 = very heavy bacterial growth). The ratio of scores of β-hemolytic coliforms to total coliforms was calculated.

### Measurement of immune response biomarkers

Whole blood samples collected on d 0, 5, and 14 PI were used for measuring total and differential blood cell counts by Comparative Pathology Laboratory at the University of California, Davis. A multiparameter, automated programmed hematology analyzer (Drew/ERBA Scientific 950 FS Hematological Analyzer, Drew Scientific Inc., Miami, FL, USA) was used for the assay to differentiate porcine blood optimally. Serum samples collected from d 0, 2, 5, and 14 PI were analyzed for C-reactive protein (CRP; R&D Systems Inc., Minneapolis, MN, USA) and haptoglobin (Aviva Systems Biology, San Diego, CA, USA) using porcine-specific enzyme-linked immunosorbent assay kits following the manufacturer’s procedures. All samples, including standards, were analyzed in duplicate. The intensity of the color was measured at 450 nm with the correction wavelength set at 530 nm using a plate reader (BioTek Instruments, Inc., Winooski, VT, USA). The concentrations of each analyte in the tested samples were calculated based on a standard curve. The intra-assay coefficients of variation for CRP and haptoglobin were 3.8% and 8.5% respectively. The inter-assay coefficients of variation for CRP and haptoglobin were 5.6% and 8.2%, respectively. Serum samples collected from d 0, 5, and 14 PI were also analyzed for inflammatory cytokines (granulocyte-macrophage colony-stimulating factor, interleukin [IL]-1α, IL-6, IL-8, and tumor necrosis factor-alpha) using a Porcine Immunology Multiplex Discovery Assay (PD13; Eve Technologies Corp, Calgary, AB, Canada).

### Statistical analysis

The normality of data was verified and outliers were identified using the UNIVARIATE procedure (SAS Institute Inc., Cary, NC, USA). Outliers were identified and removed as values that deviated from the treatment mean by more than 3 times the interquartile range. All data except frequency of diarrhea were analyzed by ANOVA using the PROC MIXED of SAS (SAS Institute Inc., Cary, NC, USA) in a randomized complete block design with the pig as the experimental unit. The statistical model included diet or time as the main effect and block as random effect. Treatment means were separated by using the LSMEANS statement and the PDIFF option of PROC MIXED. The Chi-square test was used for analyzing the frequency of diarrhea. Statistical significance and tendency were considered at *P* < 0.05 and 0.05 ≤ *P* < 0.10, respectively.

## Results

### Growth performance, diarrhea incidence, β-hemolytic coliforms

**Fig. 1:**
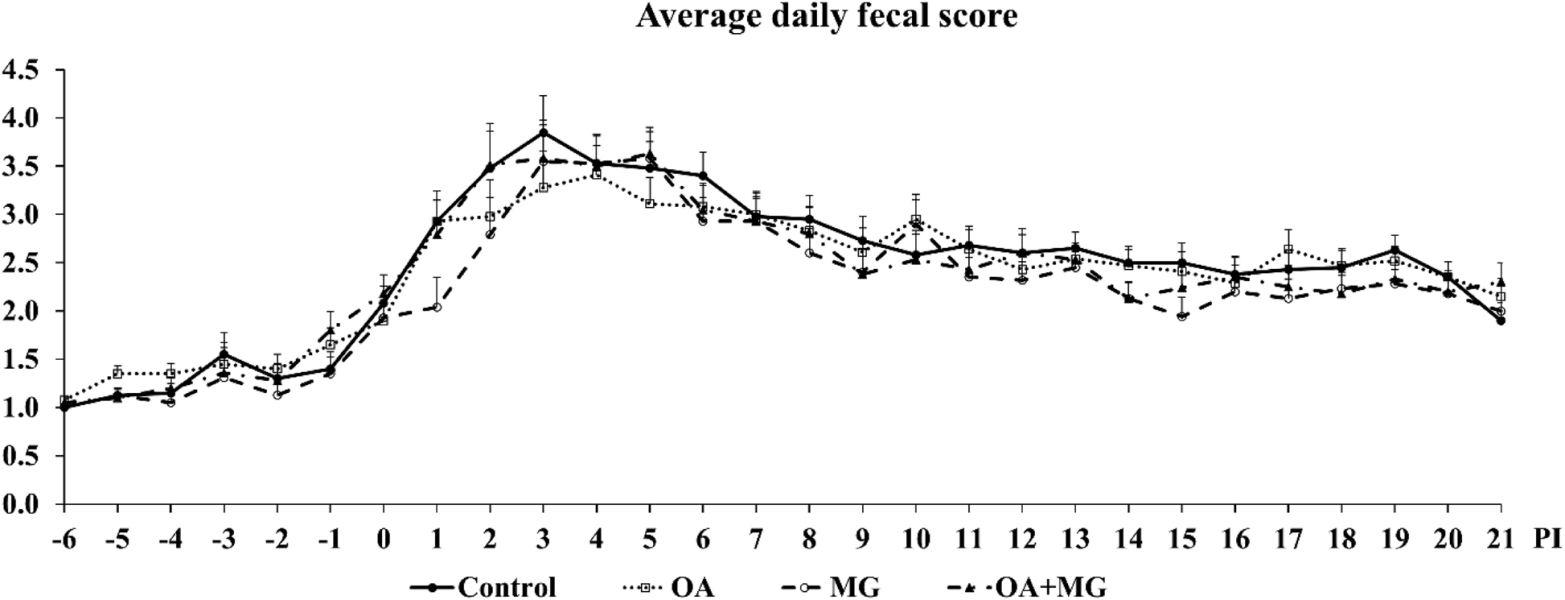
Average daily fecal score of enterotoxigenic *Escherichia coli* F18-challenged weaned pigs fed one of four experimental diets supplemented with organic acids or monoglycerides blends. Fecal score = 1, normal feces; 2, moist feces; 3, mild diarrhea; 4, severe diarrhea; 5, watery diarrhea. PI = post-inoculation. Each least squares mean represents 9 or 10 observations. OA = organic acid blend; MG = monoglyceride blend; OA + MG = combination of organic acids and monoglycerides

**Fig. 2:**
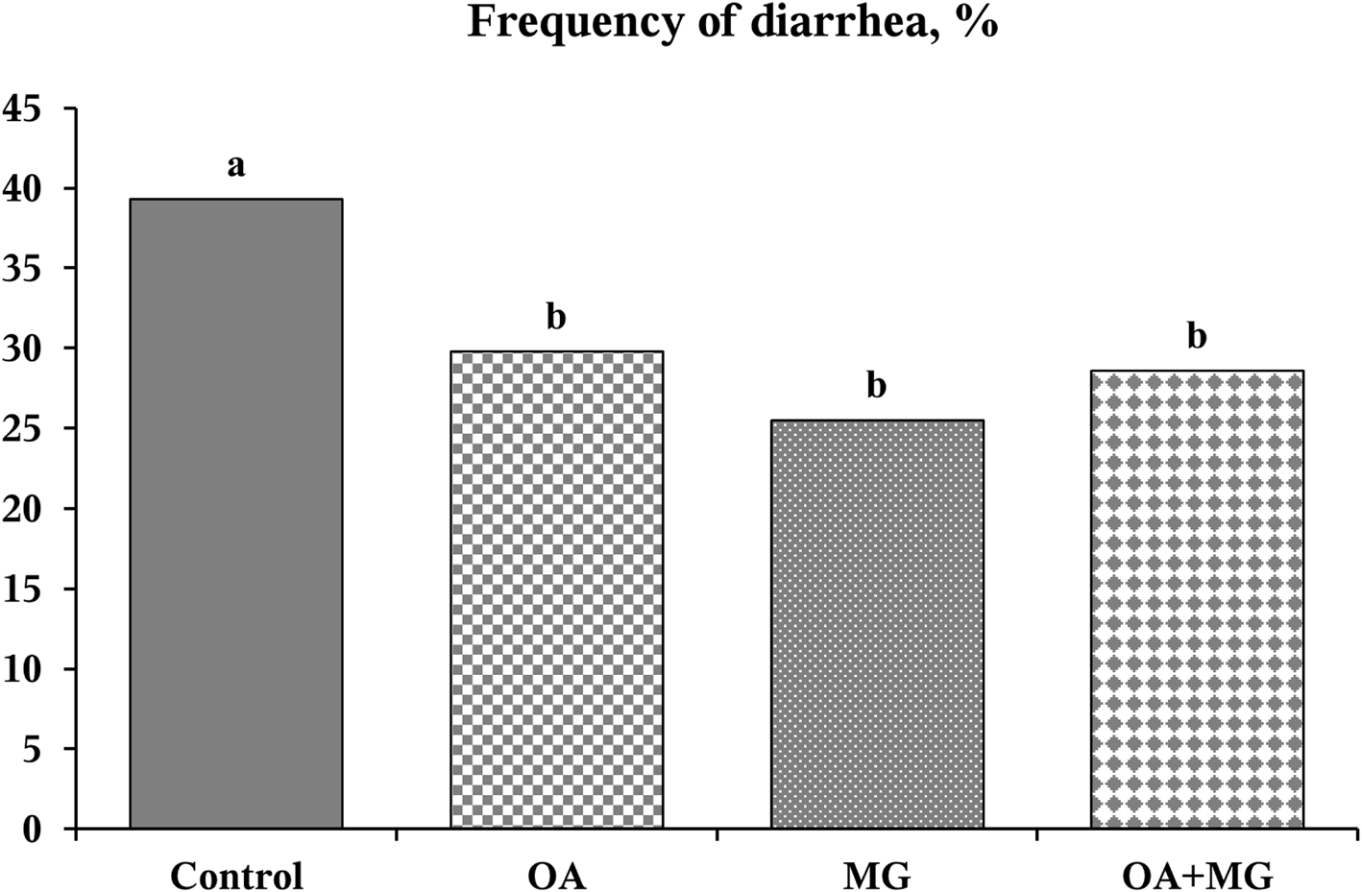
Frequency of diarrhea of enterotoxigenic *Escherichia coli* F18-challenged weaned pigs fed one of four experimental diets supplemented with organic acids or monoglycerides blends during the entire experimental period. Frequency of diarrhea was calculated as the percentage of pig days with diarrhea score ≥ 3 in the total of pig days. ^a,b^Different letters indicate significant differences (*P* < 0.05). OA = organic acid blend; MG = monoglyceride blend; OA + MG = combination of organic acids and monoglycerides

Out of 40 pigs, 1 pig in the organic acid blend group died on d 3 PI. No pigs were treated with antibiotics throughout the study. There was no significant difference (*P* > 0.05) in the initial BW of pigs among dietary treatments (Table 2). In comparison to control, supplementation of organic acids, monoglycerides, or combination did not affect BW, ADG, ADFI, and gain:feed ratio of pigs throughout the experiment. Supplementation with organic acids, monoglycerides, or combination of both did not affect average daily fecal scores throughout the experiment (Fig. 1). However, the frequency of diarrhea (fecal score ≥ 3) was lower (*P* < 0.05) in organic acids (29.77%), monoglycerides (25.49%), and combination groups (28.57%) than control group (39.29%) during the overall experimental period (Fig. 2).

**Table 2:** Growth performance of enterotoxigenic *Escherichia coli* F18-challenged weaned pigs fed one of four experimental diets supplemented with organic acids or monoglycerides blends

Item^a^	Control	OA^b^	MG^c^	OA + MG^d^	SEM	*P*-value
BW, kg						
d −7 (weaning)	7.72	7.77	7.77	7.76	0.425	0.96
d 0	9.09	8.90	9.10	8.96	0.264	0.87
d 7 PI	11.61	10.96	11.53	11.59	0.402	0.55
d 14 PI	14.83	14.23	15.07	15.02	0.602	0.73
d 21 PI	19.38	18.70	19.18	19.89	0.732	0.69
ADG, g/d						
d −7 to 0	196	154	196	167	49.3	0.48
d 0 to 7 PI	367	306	360	381	60.3	0.44
d 7 to 14 PI	459	470	509	491	47.0	0.80
d 14 to 21 PI	650	640	583	696	31.2	0.10
d 0 to 21 PI	554	554	546	593	33.5	0.71
d −7 to 21 PI	417	390	410	432	30.5	0.68
ADFI, g/d						
d −7 to 0	326	302	333	315	34.4	0.80
d 0 to 7 PI	580	553	545	569	70.9	0.95
d 7 to 14 PI	793	784	839	797	55.4	0.89
d 14 to 21 PI	1,059	1,117	1,131	1,177	69.2	0.62
d 0 to 21 PI	925	949	986	986	60.2	0.83
d −7 to 21 PI	690	691	712	715	52.0	0.95
Gain:Feed						
d −7 to 0	0.59	0.50	0.58	0.48	0.101	0.31
d 0 to 7 PI	0.61	0.56	0.66	0.66	0.050	0.29
d 7 to 14 PI	0.55	0.60	0.61	0.62	0.034	0.46
d 14 to 21 PI	0.62	0.58	0.52	0.59	0.035	0.08
d 0 to 21 PI	0.60	0.58	0.56	0.60	0.028	0.40
d −7 to 21 PI	0.60	0.57	0.58	0.60	0.020	0.57

^a^*BW* Body weight, *ADG* Average daily gain, *ADFI* Average daily feed intake, *PI* Post-inoculation. Each least squares mean represents 9 or 10 observations

^b^*OA* Organic acid blend

^c^*MG* Monoglyceride blend

^d^*OA* + *MG* Combination of organic acids and monoglycerides

**Fig. 3:**
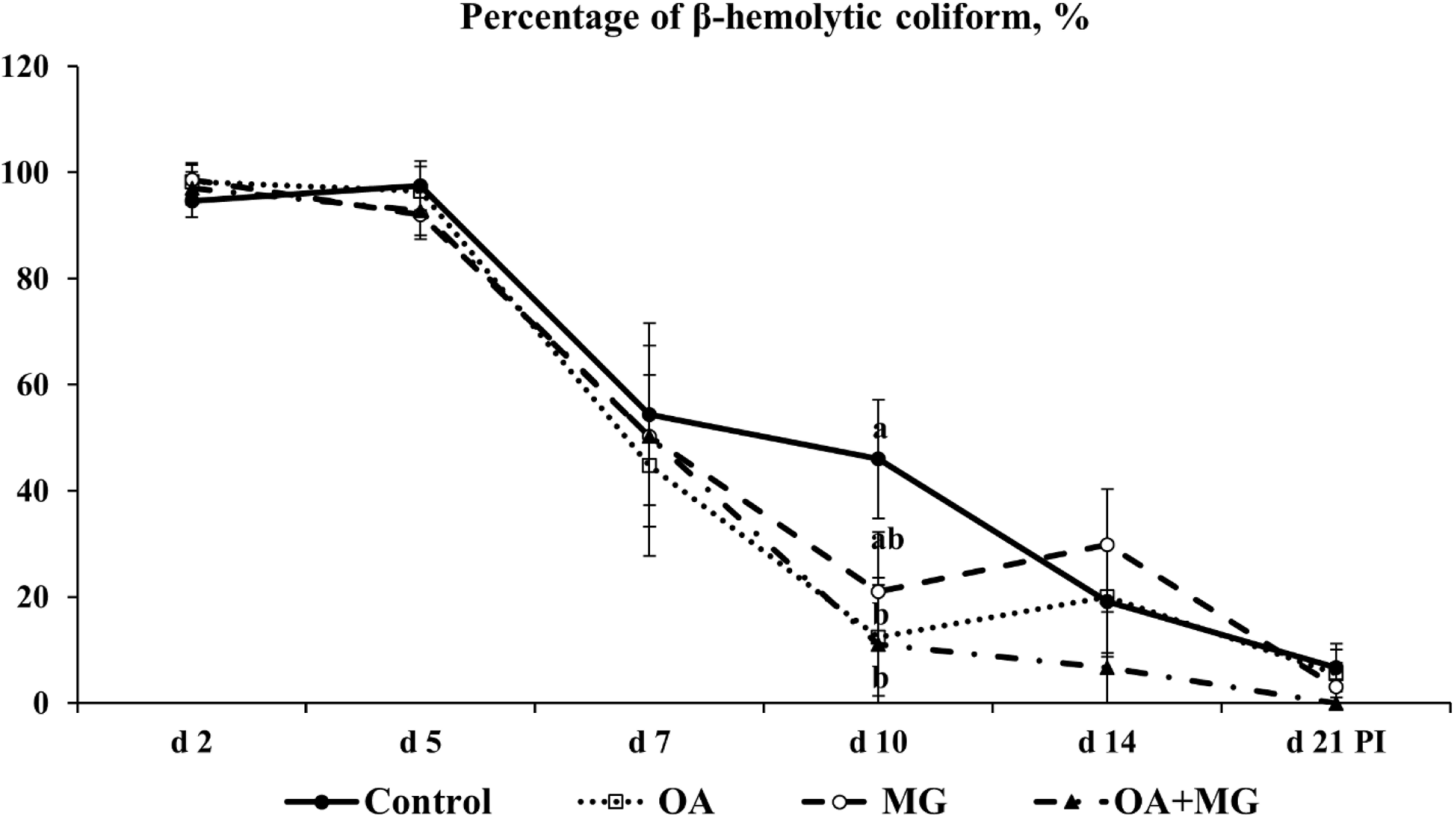
The percentage (%) of β-hemolytic coliform in fecal samples of enterotoxigenic *Escherichia coli* F18-challenged pigs fed one of four experimental diets supplemented with organic acids or monoglycerides blends. Each least squares mean represents 9 or 10 observations. ^a,b^Different letters indicate significant differences (*P* < 0.05). OA = organic acid blend; MG = monoglyceride blend; OA + MG = combination of organic acids and monoglycerides

No β-hemolytic coliforms were identified in fecal samples of pigs before ETEC F18 inoculation, but the β-hemolytic coliforms were detected in feces from all pigs on d 2 PI (Fig. 3). No significant differences were observed in the percentage of fecal β-hemolytic coliforms at each time point, except that organic acid blend and combination reduced (*P* < 0.05) the percentage of fecal β-hemolytic coliforms on d 10 PI compared to control. However, comparable percentages of β-hemolytic coliforms in feces were observed in the three supplemented groups on d 10 PI.

### Bacterial translocation

**Fig. 4:**
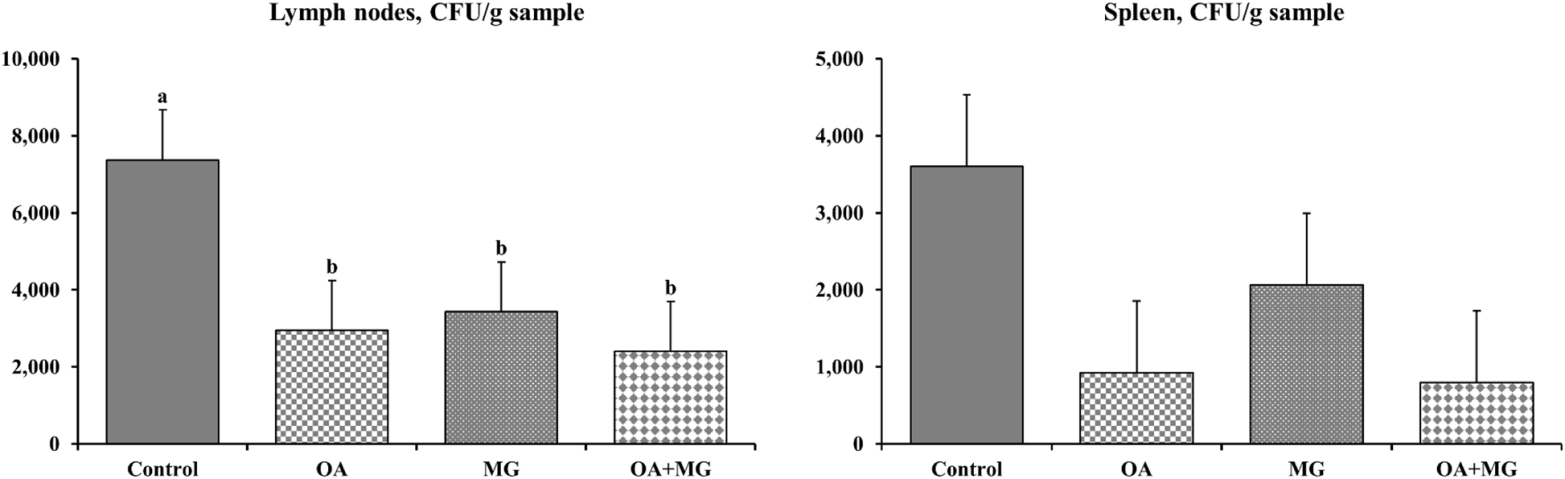
Total coliforms in mesenteric lymph nodes and spleen of enterotoxigenic *Escherichia coli* F18-challenged weaned pigs fed one of 4 experimental diets supplemented with organic acids or monoglycerides blends. ^a,b^Different letters indicate significant differences (*P* < 0.05). Each least squares mean represents 9 or 10 observations. OA = organic acid blend; MG = monoglyceride blend; OA + MG = combination of organic acids and monoglycerides

Pigs supplemented with organic acids, monoglycerides, or combination had lower (*P* < 0.05) counts of total coliforms (CFU/g) in mesenteric lymph nodes on d 21 PI compared with control (Fig. 4). The total coliforms (CFU/g) in mesenteric lymph nodes did not differ between the three supplemented groups. There was no significant difference in total coliforms (CFU/g) in spleen among the dietary treatments.

### Systemic immunity and red blood cell profile

Irrespective of diet, the ETEC F18 inoculation increased (*P* < 0.05) the total white blood cell (WBC) number, and the counts of neutrophils, lymphocytes, monocytes, eosinophils, and basophils as the pig age increased (Table 3). The proportion of eosinophils and basophils was increased (*P* < 0.05) on d 14 PI compared with d 0 or 5 PI. The ETEC F18 infection also affected the RBC profile by decreasing (*P* < 0.05) red cell distribution width (RDW) on d 5 and 14 PI, but increasing (*P* < 0.05) the percentage of packed cell volume (hematocrit) and levels of hemoglobin and total protein on d 14 PI.

**Table 3:** Total and differential white blood cells and red blood profile in weaned pigs throughout the experiment

Item^d^	d 0	d 5 PI	d 14 PI	SEM	*P*-value
WBC, 10^3^/μL	7.81^c^	10.09^b^	11.95^a^	0.441	< 0.01
Neu, 10^3^/μL	3.69^b^	5.09^a^	5.26^a^	0.313	< 0.01
Lym, 10^3^/μL	3.34^c^	4.08^b^	4.96^a^	0.224	< 0.01
Mono, 10^3^/μL	0.53^c^	0.78^b^	0.94^a^	0.050	< 0.01
Eos, 10^3^/μL	0.15^b^	0.19^b^	0.55^a^	0.051	< 0.01
Baso, 10^3^/μL	0.01^b^	0.02^a^	0.02^a^	0.002	< 0.01
Neu, %	46.07	48.96	44.99	1.549	0.20
Lym, %	43.06	40.72	41.73	1.529	0.54
Mono, %	7.13	7.47	8.09	0.466	0.29
Eos, %	1.89^b^	1.54^b^	4.89^a^	0.494	< 0.01
Baso, %	0.13^b^	0.15^ab^	0.18^a^	0.017	< 0.05
Neu:Lym	1.03	1.19	1.06	0.079	0.18
RBC, 10^6^/μL	7.38	7.34	7.55	0.145	0.39
HGB, g/dL	8.16^b^	8.11^b^	8.68^a^	0.283	< 0.05
HCT, %	30.39^b^	29.72^b^	32.57^a^	0.554	< 0.01
MCV, fL^e^	41.58^ab^	40.66^b^	42.70^a^	0.815	< 0.01
MCH, pg	11.35	11.16	11.78	0.509	0.22
MCHC, g/dL	27.16	27.01	27.43	0.719	0.88
RDW, %	29.23^a^	27.29^b^	26.31^b^	0.673	< 0.01
Platelets, 10^3^/μL	461	441	421	57.2	0.42
MPV, fL^e^	7.7	7.5	7.6	0.21	0.21
Total protein, g/dL	4.98^b^	4.94^b^	5.15^a^	0.078	< 0.05

^a–c^Values without a common superscript are different (*P* < 0.05)

^d^*WBC* White blood cell, *Neu* Neutrophil, *Lym* Lymphocyte, *Mono* Monocyte, *Eos* Eosinophil, *Baso* Basophil, *RBC* Red blood cell, *HGB* Hemoglobin, *HCT* Hematocrit (Packed cell volume), *MCV* Mean corpuscular volume, *MCH* Mean corpuscular hemoglobin, *MCHC* Mean corpuscular hemoglobin concentration, *RDW* Red cell distribution width, *MPV* Mean platelet volume. Each least squares mean represents 39 or 40 observations

^e^*fL* Femtolitre (10^–15^ L)

No difference was observed in the WBC profile among dietary treatments on d 0 before ETEC F18 inoculation (Table 4). Supplementation of organic acids did not affect the WBC profiles on d 5 and 14 PI. Pigs fed with monoglycerides or combination had lower (*P* < 0.05) WBC counts on d 5 PI, compared with control. Supplementation of combination also reduced (*P* < 0.05) number of lymphocytes and neutrophil-to-lymphocyte ratio (NLR) on d 5 PI compared with control. However, pigs supplemented with monoglycerides had greater (*P* < 0.05) numbers of WBC and neutrophils and higher (*P* < 0.05) NLR on d 14 PI compared to control.

**Table 4:** Total and differential white blood cells in enterotoxigenic *Escherichia coli* F18-challenged weaned pigs fed one of four experimental diets supplemented with organic acids or monoglycerides blends

Item^c^	Control	OA^d^	MG^e^	OA + MG^f^	SEM	*P*-value
d 0 before inoculation						
WBC, 10^3^/μL	8.16	7.68	7.68	7.74	0.437	0.91
Neu, 10^3^/μL	3.91	3.51	3.56	3.77	0.298	0.92
Lym, 10^3^/μL	3.47	3.42	3.24	3.21	0.221	0.78
Mono, 10^3^/μL	0.63	0.46	0.54	0.48	0.092	0.21
Eos, 10^3^/μL	0.113	0.135	0.187	0.175	0.095	0.67
Baso, 10^3^/μL	0.01	0.01	0.01	0.01	0.004	0.95
Neu, %	46.30	44.97	46.26	46.77	3.016	0.97
Lym, %	43.72	43.05	41.90	43.56	2.887	0.97
Mono, %	8.03	6.14	8.00	6.37	0.862	0.28
Eos, %	1.38	1.65	2.39	2.14	0.911	0.63
Baso, %	0.11	0.14	0.15	0.12	0.031	0.89
Neu:Lym	1.11	0.95	1.07	0.97	0.079	0.70
d 5 post-inoculation						
WBC, 10^3^/μL	11.59^a^	10.19^ab^	9.86^b^	8.72^b^	0.461	< 0.05
Neu, 10^3^/μL	5.83	5.32	4.79	4.41	0.314	0.33
Lym, 10^3^/μL	4.66^a^	3.87^ab^	4.11^ab^	3.68^b^	0.228	< 0.05
Mono, 10^3^/μL	0.89	0.69	0.83	0.71	0.097	0.36
Eos, 10^3^/μL	0.193	0.257	0.170	0.154	0.095	0.63
Baso, 10^3^/μL	0.02	0.02	0.02	0.02	0.005	0.98
Neu, %	50.08	51.48	48.84	45.46	3.179	0.30
Lym, %	40.40	39.75	39.82	42.89	3.043	0.66
Mono, %	7.63	6.28	8.30	7.66	0.908	0.33
Eos, %	1.28	1.51	1.59	1.77	0.964	0.89
Baso, %	0.12	0.15	0.18	0.13	0.035	0.41
Neu:Lym	1.32^a^	1.20^ab^	1.26^a^	0.99^b^	0.081	< 0.05
d 14 post-inoculation						
WBC, 10^3^/μL	10.29^b^	12.20^ab^	12.74^a^	12.57^ab^	0.456	< 0.05
Neu, 10^3^/μL	4.09^b^	5.48^ab^	6.33^a^	5.15^ab^	0.313	< 0.05
Lym, 10^3^/μL	4.86	5.32	4.67	4.97	0.224	0.77
Mono, 10^3^/μL	0.94	0.86	1.05	0.93	0.102	0.72
Eos, 10^3^/μL	0.418	0.529	0.547	0.716	0.090	0.50
Baso, 10^3^/μL	0.02	0.02	0.03	0.02	0.004	0.67
Neu, %	42.91	42.49	51.12	43.45	3.016	0.33
Lym, %	44.05	41.94	37.74	43.18	3.227	0.62
Mono, %	8.71	8.15	7.98	7.51	1.028	0.85
Eos, %	4.18	4.35	5.34	5.68	0.866	0.79
Baso, %	0.16	0.15	0.23	0.19	0.033	0.34
Neu:Lym	0.89^b^	0.94^ab^	1.36^a^	1.04^ab^	0.079	< 0.05

^a,b^Values without a common superscript are different (*P* < 0.05)

^c^*WBC* White blood cell, *Neu* Neutrophil, *Lym* Lymphocyte, *Mono* Monocyte, *Eos* Eosinophil, *Baso* Basophil. Each least squares mean represents 9 or 10 observations

^d^*OA* Organic acid blend

^e^*MG* Monoglyceride blend

^f^*OA* + *MG* Combination of organic acids and monoglycerides

The effects of dietary supplementation of organic acids, monoglycerides, or combination on the RBC profiles of weaned pigs were limited on d 0, with the exception that pigs in organic acid blend group had increased (*P* < 0.05) mean platelet volume (Table 5), which continued into the subsequent periods. Pigs in the combination group had greatest (*P* < 0.05) RBC on d 5 PI. On d 14 PI, pigs in the combination group had greatest (*P* < 0.05) RBC and hemoglobin counts, but lowest (*P* < 0.05) platelet counts among dietary treatments.

**Table 5:** Red blood cell profile in enterotoxigenic *Escherichia coli* F18-challenged weaned pigs fed one of four experimental diets supplemented with organic acids or monoglycerides blends

Item^d^	Control	OA^e^	MG^f^	OA + MG^g^	SEM	*P*-value
d 0 before inoculation						
RBC, 10^6^/μL	7.22	7.36	7.38	7.54	0.241	0.78
HGB, g/dL	8.22	7.89	8.29	8.24	0.465	0.80
HCT, %	29.19	30.70	30.68	31.00	1.068	0.56
MCV, fL^h^	41.51	42.18	41.46	41.18	1.121	0.78
MCH, pg	11.42	11.76	11.26	10.96	0.674	0.66
MCHC, g/dL	27.38	27.68	27.09	26.50	1.015	0.77
RDW, %	29.11	29.32	29.27	29.23	1.067	0.98
Platelets, 10^3^/μL	482	522	396	443	69.1	0.32
MPV, fL^h^	7.5^b^	8.2^a^	7.5^b^	7.7^ab^	0.21	< 0.05
Total protein, g/dL	5.01	5.08	4.89	4.96	0.116	0.46
d 5 post-inoculation						
RBC, 10^6^/μL	7.26^ab^	7.04^b^	7.33^ab^	7.76^a^	0.253	< 0.05
HGB, g/dL	8.14	7.66	8.23	8.43	0.487	0.65
HCT, %	29.61	28.50	29.44	31.36	1.068	0.37
MCV, fL^h^	40.98	40.80	40.16	40.72	1.163	0.86
MCH, pg	11.23	11.16	11.22	11.02	0.697	0.99
MCHC, g/dL	27.77	27.13	27.85	26.89	1.103	0.74
RDW, %	27.06	27.84	26.64	27.62	1.118	0.85
Platelets, 10^3^/μL	384	494	441	443	73.3	0.37
MPV, fL^h^	7.5^ab^	7.9^a^	7.3^b^	7.5^ab^	0.21	< 0.05
Total protein, g/dL	4.83	5.07	4.99	4.86	0.121	0.46
d 14 post-inoculation						
RBC, 10^6^/μL	7.23^b^	7.20^b^	7.62^ab^	8.16^a^	0.241	< 0.05
HGB, g/dL	8.68^ab^	8.01^b^	9.05^ab^	9.69^a^	0.465	< 0.05
HCT, %	31.74^bc^	29.53^c^	33.24^ab^	35.75^a^	1.068	< 0.05
MCV, fL^h^	42.73	41.23	42.86	44.00	1.121	0.54
MCH, pg	12.05	11.28	11.84	11.95	0.674	0.86
MCHC, g/dL	27.97	26.85	27.89	27.03	1.055	0.81
RDW, %	26.74	27.04	25.24	26.20	1.118	0.88
Platelets, 10^3^/μL	509^a^	434^ab^	409^ab^	331^b^	67.6	< 0.05
MPV, fL^h^	7.7^ab^	8.1^a^	7.3^b^	7.4^b^	0.21	< 0.05
Total protein, g/dL	5.14	5.05	5.30	5.08	0.116	0.29

^a–c^Values without a common superscript are different (*P* < 0.05)

^d^*RBC* Red blood cell, *HGB* Hemoglobin, *HCT* Hematocrit (Packed cell volume), *MCV* Mean corpuscular volume, *MCH* Mean corpuscular hemoglobin, *MCHC* Mean corpuscular hemoglobin concentration, *RDW* Red cell distribution width, *MPV* Mean platelet volume. Each least squares mean represents 9 or 10 observations

^e^*OA* Organic acid blend

^f^*MG* Monoglyceride blend

^g^*OA* + *MG* Combination of organic acids and monoglycerides

^h^*fL* Femtolitre (10^–15^ L)

Pigs in the monoglycerides group tended (*P* < 0.10) to have the lowest IL-1α level on d 0 and 14 PI, followed by pigs in the combination group (Table 6). However, dietary treatments did not affect IL-6 and IL-8 levels. Supplementation of monoglycerides also tended (*P* < 0.10) to reduce serum granulocyte-macrophage colony-stimulating factor and tumor necrosis factor-alpha on d 0 and 5 PI. No difference was observed in C-reactive protein and haptoglobin levels among dietary treatments throughout the experiment.

**Table 6:** Serum inflammatory cytokines and acute phase proteins in enterotoxigenic *Escherichia coli* F18-challenged weaned pigs fed one of four experimental diets supplemented with organic acids or monoglycerides blends

Item^c^	Control	OA^d^	MG^e^	OA + MG^f^	SEM	*P*-value
d 0 before inoculation						
GM-CSF, pg/mL	97.63^a^	66.40^ab^	19.88^b^	106.11^a^	41.781	0.06
IL-1α, pg/mL	136.68^a^	142.19^a^	17.35^b^	65.23^ab^	63.873	0.07
IL-6, pg/mL	120.02	183.34	83.06	207.92	83.487	0.52
IL-8, pg/mL	140.32	125.81	136.91	177.77	25.036	0.29
TNF-α, pg/mL	228.58^ab^	136.31^ab^	49.05^b^	302.93^a^	88.085	0.056
C-reactive protein, µg/mL	6.77	4.32	4.62	6.13	1.539	0.63
Haptoglobin, mg/mL	1.70	1.40	0.99	1.42	0.470	0.24
d 2 post-inoculation						
C-reactive protein, µg/mL	17.85	19.55	18.13	16.99	4.954	0.93
Haptoglobin, mg/mL	2.16	2.82	2.14	2.23	0.662	0.60
d 5 post-inoculation						
GM-CSF, pg/mL	51.06^ab^	53.02^ab^	27.57^b^	88.94^a^	22.394	0.055
IL-1α, pg/mL	138.70	152.51	31.53	62.66	66.989	0.12
IL-6, pg/mL	148.57	209.48	117.35	213.25	90.719	0.76
IL-8, pg/mL	106.33	150.79	110.00	211.33	39.635	0.12
TNF-α, pg/mL	129.86	141.61	31.45	194.62	67.521	0.18
C-reactive protein, µg/mL	7.81	9.01	10.24	8.27	2.065	0.79
Haptoglobin, mg/mL	1.43	1.67	1.39	0.97	0.679	0.57
d 14 post-inoculation						
GM-CSF, pg/mL	48.18	47.16	32.45	50.28	6.582	0.22
IL-1α, pg/mL	103.34^ab^	148.50^a^	27.40^b^	47.13^b^	67.268	0.07
IL-6, pg/mL	83.62	169.12	101.47	145.67	60.162	0.68
IL-8, pg/mL	96.90	108.91	93.83	171.07	49.015	0.10
TNF-α, pg/mL	81.19	54.00	29.66	76.27	22.408	0.35
C-reactive protein, µg/mL	7.59	8.17	11.28	9.01	2.328	0.69
Haptoglobin, mg/mL	0.83	0.94	0.88	0.54	0.394	0.74

^a,b^Values without a common superscript are different (*P* < 0.05)

^c^*GM-CSF* Granulocyte–macrophage colony-stimulating factor, *IL-1α* Interleukin-1 alpha, *IL-6* Interleukin 6, *IL-8* Interleukin 8, *TNF*-*α* Tumor necrosis factor-alpha. Each least squares mean represents 9 or 10 observations

^d^*OA* Organic acid blend

^e^*MG* Monoglyceride blend

^f^*OA* + *MG* Combination of organic acids and monoglycerides

## Discussion

In the swine industry, post-weaning diarrhea caused by ETEC F18 is a significant disease in terms of the negative impact on productivity, profitability, and sustainability, particularly due to issues related to antibiotic resistance and environmental impacts [40, 41]. Despite growing interest in the bioactivity and effects of nutritional interventions such as organic acids and monoglycerides, there has been limited research on disease resistance and resilience in weaned pigs under disease challenge conditions. The present study aims to contribute to our understanding of practical nutritional intervention by investigating the impacts of various mixtures of acid-based feed additives on performance, diarrhea, fecal culture, bacterial translocation, and systemic inflammation of weaned pigs challenged with ETEC F18.

ETEC with the F18 fimbrial adhesin, the primary cause of post-weaning diarrhea, adheres to and proliferates in the small intestine, producing enterotoxins that disrupt fluid homeostasis [5, 42, 43]. Diarrhea, bacterial fecal shedding, and associated signs are commonly used to assess challenge efficacy [44]. Following ETEC F18 inoculation, increased diarrhea and β-hemolytic coliform fecal shedding were observed, indicating successful infection in our challenge model. These observations are consistent with our previous studies using the same ETEC strain, which also showed peak infection on d 3 to 5 PI followed by gradual recovery [32, 37, 45]. The current study demonstrates that supplementation with organic acid blend, monoglyceride blend, or their combination effectively reduced the frequency of diarrhea in ETEC F18-infected pigs. This is supported by changes in the percentage of β-hemolytic coliform in feces. While there was no difference in β-hemolytic coliform fecal shedding among the treatment groups during the early infection stage, pigs in the organic acids and combination groups exhibited the lowest fecal shedding on d 10 PI, followed by pigs in the monoglycerides group. These findings are consistent with previous research suggesting that the reduction in diarrhea of pigs may be due to the inhibition of ETEC proliferation by supplementing organic acids [46], monoglycerides [47], or combination [48].

Organic acids have unique pKa values ​​and exhibit different degrees of dissociation depending on the pH of their environment. The undissociated form can penetrate bacterial cell membranes, lowering intracellular pH and disrupting bacterial homeostasis [13]. Combinations of acids with different pKa values can exert broad effects throughout the gastrointestinal tract [49, 50]. Monoglycerides, on the other hand, act pH-independently [51, 52] and primarily disrupt bacterial phospholipid membranes, altering membrane permeability and exerting antibacterial effects [51, 53]. Synergistic antibacterial effect of the combination of organic acids and monoglycerides has been reported in in vitro microbiology studies [28, 29]. In the current in vivo challenge study, both organic acids and monoglycerides reduced the frequency of diarrhea and fecal shedding of β-hemolytic coliforms, but no additional reduction was observed in the combination group. The results of diarrhea frequency and fecal shedding suggest that continuous supplementation with mixtures of acid-based additives has the potential to enhance intestinal barrier function and resilience to pathogenic challenges over time.

To investigate the effects of organic acids, monoglycerides, and their combination on intestinal barrier function and immune responses in ETEC-infected pigs, bacterial translocation and systemic immunity were examined. Regardless of the harmfulness of bacteria in the gut, bacterial invasion into the other organs due to bacterial overgrowth, intestinal barrier dysfunction, or compromised immune system can lead to systemic inflammation and damage [54]. This invasion is often observed in disease conditions and can serve as an indicator of bacterial infection [54, 55]. Different bacteria may vary in their efficiency of translocating from the gastrointestinal tract to organs, such as mesenteric lymph nodes, spleen, and liver. Mesenteric lymph nodes are particularly efficient site for translocating Enterobacteriaceae family (Gram-negative, facultatively anaerobic; e.g., *Escherichia coli*) [55, 56]. The results of bacterial translocation indicated that the organic acid blend, monoglyceride blend, and their combination improved the integrity of the intestinal barrier during ETEC infection, potentially reducing systemic spread of pathogens.

The activation of innate immunity by lipopolysaccharides (LPS) in the outer membrane of ETEC leads to the release of inflammatory cytokines, immune cell recruitment, and disruption of intestinal integrity, resulting in systemic inflammation [5, 41]. The increase in WBC counts observed in this study aligns with previous research [32, 47, 57], indicating that innate immunity plays a significant role in both the early stages as well as throughout the progression of ETEC infection, reflecting a systemic inflammatory response. Supplementation with monoglycerides or the combination reduced WBC counts on d 5 PI, suggesting modulation of the inflammatory response. Moreover, although the blend of organic acid or monoglyceride groups showed intermediate levels, the combination group exhibited lower lymphocytes and NLR than the control group, indicating enhanced immune-modulatory effects in ETEC F18-challenged pigs fed the combination near the peak of infection. These findings are in close agreement with our previous research involving other types of acid-based products (i.e., monobutyrin or monovalerin) [47], suggesting that supplementation with the combination may synergistically reduce systemic inflammation induced by ETEC infection. Our observations of reduced inflammatory cytokines further support the immunomodulatory effects of monoglycerides.

Interestingly, monoglycerides supplementation further increased WBC, neutrophils, and NLR on d 14 PI, potentially linked to the activation of G protein-coupled receptors (GPCR) by monoglyceride-derived MCFA. It has been reported that MCFA act as ligands for various GPCR present in the plasma membrane of various cells, influencing cellular processes [58]. Specifically, GPR84, predominantly expressed on immune cells, enhances the inflammatory response upon activation [59–61] and has been reported to be upregulated by LPS [59, 60]. Therefore, the relatively high level of neutrophils in the monoglycerides group on d 14 PI may be associated with sustained innate immune response [62–64], contributing to pathogen clearance.

Despite the aforementioned symptomatic and physiological improvements, there were no differences across treatments in the growth performance of newly weaned pigs challenged with ETEC F18 in this study. These findings are also consistent with previous studies where the addition of acid-based additives improved intestinal and immune status but did not significantly impact growth performance [26, 65]. The relatively small number of replicates per treatment and individual housing in the current study may also limit the detection of performance differences among treatments. It is important to note that enhancing overall disease resistance in pigs, particularly during the post-weaning period, may reduce the risk of other enteric diseases and the necessity for antibiotic use.

## Conclusions

In summary, the present study thoroughly investigated the effects of several mixtures of acid-based feed additives on the performance, diarrhea, fecal shedding, bacterial translocation, and immunity of newly weaned pigs challenged with ETEC F18. Supplementation with organic acid blend containing, among others, formic acid, a monoglyceride blend of short- and medium-chain fatty acids, or a combination of both acid-based additives benefited the control of post-weaning diarrhea and may alleviate intestinal damage caused by ETEC infection as indicated by reduced fecal shedding and bacterial translocation. The monoglyceride blend exhibited immunomodulatory effects on the host and shows promise for synergistic effects with organic acids, warranting further investigation.

## Data Availability

All data generated or analyzed during this study are available from the corresponding author upon reasonable request.

## Abbreviations

ADFI: Average daily feed intake
ADG: Average daily gain
BW: Body weight
CFU: Colony-forming units
CRP: C-reactive protein
ETEC: Enterotoxigenic *Escherichia coli*
GPCR: G protein-coupled receptors
GRAS: Generally recognized as safe
IL: Interleukin
LPS: Lipopolysaccharides
MCFA: Medium-chain fatty acids
NLR: Neutrophil-to-lymphocyte ratio
PI: Post-inoculation
RBC: Red blood cell
RDW: Red cell distribution width
WBC: White blood cell
